# O-06 - FORECASTING HEALTH PROTECTION OPERATIONAL WORKLOAD IN NORTHERN IRELAND USING NEGATIVE BINOMIAL GENERALISED ADDITIVE MODELS

**DOI:** 10.1093/pubmed/fdag046.006

**Published:** 2026-07-09

**Authors:** Ms Sarah Arnold, Ms Fiona Morris, Ms Kelly Neville, Dr Declan Bradley

**Affiliations:** Public Health Agency; Public Health Agency; Public Health Agency; Public Health Agency

## Abstract

**Background:**

The health protection team in the Public Health Agency in Northern Ireland is responsible for assessing and responding to cases, outbreaks and enquiries relating to infectious diseases and other hazards to public health. Short-term forecasting of operational workload would support staffing and resource planning. The COVID-19 pandemic caused significant changes in health protection activity, complicating the use of historical data for forecasting.

**Objectives:**

To develop and validate an operational 14-day forecast of four health protection response streams (cases, contacts, enquiries and situations) using routinely collected HP Zone data.

**Methods:**

Negative binomial generalised additive models (GAMs) were fitted for each outcome, incorporating long-term trend splines, cyclic day-of-year seasonality, day-of-week effects, bank holiday indicators and autoregressive lag terms. The COVID-19 period (March 2020-March 2023) was excluded from training windows. Training windows were selected empirically using rolling origin cross-validation. Forecast uncertainty was quantified using 80% prediction intervals. Models were validated on holdout data across pre-COVID, COVID and post-COVID periods.

**Results:**

All four models demonstrated adequate fit and acceptable holdout performances across pre-COVID and post-COVID validation periods. The cases model explained 75.8% of deviance while the enquiries model explained 81%. Residual diagnostics confirmed distributional assumptions were met. The pipeline produces 14-day forecasts with prediction intervals that are updated automatically each day.

**Conclusions:**

Negative binomial GAMs provide a viable and interpretable framework for forecasting health protection operational workload, demonstrating the feasibility of embedding short-term forecasting into routine business and staffing planning for health protection. Next steps include testing the pipeline in practice and refining it based on feedback from users.

